# Birth and death processes in phylogenetics and population genetics

**DOI:** 10.1098/rstb.2023.0300

**Published:** 2025-02-20

**Authors:** Simon Tavaré

**Affiliations:** ^1^ Irving Institute for Cancer Dynamics, Columbia University, 1190 Amsterdam Avenue, New York, NY 10027, USA

**Keywords:** statistical inference, marked Poisson processes, coalescent, phylogenetic trees, approximate Bayesian computation, Yule process, distributional random forests

## Abstract

This review focuses on linear birth-and-death processes (LBDPs), describing the basic properties of the population-size process and the underlying ancestral trees that record how the evolving species (or individuals or cells) are related. The first section describes the Yule, or linear birth, process setting. Analogous results for the birth-and-death process (BDP) are given. The stochastic structure of the reconstructed tree obtained by pruning branches that do not survive to the present time is detailed. In §2, the BDP with immigration is described. Immigration is a mechanism to introduce new types into a population evolving through time. For the Yule process, marked Poisson process arguments are used to illustrate properties of the sample variance of the number of families observed in two consecutive time intervals. In the final section, we describe a recent method for approximate Bayesian computation using random forests, and illustrate it with an example of inference from DNA sequence data about the split rate and mutation rate in a birth-and-death model for the evolution of a cell population.

This article is part of the theme issue ‘“A mathematical theory of evolution”: phylogenetic models dating back 100 years’.

## 1. Introduction

The birth-and-death processes (BDPs) are a family of Markovian stochastic models for the evolution through time of the number of species in a genus (or individuals in a population, or copies of an allele, or …). At each event in the process, the number of individuals increases by one, or decreases by one unless the process has already died out.

We denote by Z(t) the population size at time t≥0 and the state space by N:={0,1,2,3,…}. The evolution is usually described by the infinitesimal probabilities of changes to the population size:


$$P(Z(t+h)=m|Z(t)=n)=\left\{ \begin{array}{cc} {\text{λ}}_{n}(t)h+o(h) & m=n+1,\\ \mu_{n}(t)h+o(h) & m=n-1,\\ 1-({\text{λ}}_{n}(t)+\mu_{n}(t))h+o(h) & m=n \end{array}\right.$$

as h→0. When either of the rates ${\text{λ}}_{n}(t)$ or $\mu_{n}(t)$ depends on t, the process is said to be time-inhomogeneous and otherwise homogeneous. The stochastic behaviour of the general time-homogenous case—determining transience, recurrence, extinction probabilities, stationary distributions and so on—is discussed in many elementary textbooks (e.g. [1], Chapter 4). We won’t discuss this further here.

In phylogenetics and population genetics, the rates are often taken to be linear and are given by


(1.1) $${\text{λ}}_{n}(t)=n\text{λ}(t),\;\mu_{n}(t)=n\mu (t)$$

so that λ(t) and μ(t) are the *per capita* birth and death rates at time t. Time-homogeneous linear BDPs were studied by Feller [2] and the time-inhomogeneous models by Kendall [3]. These processes are a special case of the Markov branching process, for which the offspring distribution allows for more than a single birth at a time ([1], Chapter 8).

In this article, we discuss a tiny fraction of the literature on linear BDPs (LBDPs) with rates given by equation (1.1), beginning with the motivating article for this special issue: the Yule (or pure birth) process [4], which has μ(t)≡0. After a brief description of the stochastic properties of Yule’s model, we will describe some properties of the LBDP, including a discussion of the underlying tree structure of the process, be it the complete phylogenetic tree or a reconstructed one in which the history of the extant species is recovered. For ease of exposition, we concentrate on the homogeneous case below.

In §4 we use a Poisson immigration process as a way to introduce new families (or genera, or alleles or types or …) into the model, and show how elementary marked Poisson process arguments may be used to derive a number of quantities of interest about the different families. We illustrate the results with a celebrated example from population genetics, the Ewens sampling formula [5], and then consider a more ecological example that addresses Fisher *et al*.’s [6] multiple sampling problem.

In §5 we discuss aspects of statistical inference for these models. Here, the intention is to illustrate some recent developments in approximate Bayesian computation (ABC) in settings where the methods may be validated, but which hold great promise for more complex models in which likelihoods are either intractable or very time consuming to compute.

## 2. The Yule process

### (a) Distributional properties

We consider a Yule process starting from a single species in a genus, and suppose that new species arise at constant rate *λ*, independently from any existing species. We let $T_{1},T_{2},T_{3}\ldots$ denote the time during which there is a single species, two species, three species and so on. $T_{j}$ has an exponential distribution with parameter jλ, since $T_{j}$ is the minimum of j independent exponential random variables, one for each species currently alive. The distribution of Z(t) was originally derived via a differential equation (cf. [7]), but we will use a different approach (cf. [8]) that is useful in the sequel.

We find the joint density $f_{n}$ of $U_{1}=T_{1},U_{2}=T_{1}+T_{2},\ldots ,U_{n-1}=T_{1}+\cdots +T_{n-1}$ as follows. For $u_{0}=0<u_{1}<\cdots <u_{n-1}<t$, we see that the genus has n species at time t and split times $u_{1}<\cdots <u_{n-1}$ with density


(2.1) $$\begin{array}{rl} f_{n}(u_{1},\ldots ,u_{n-1})\; & =\;\text{λ}e^{-\text{λ}u_{1}}\cdot 2\text{λ}e^{-2\text{λ}(u_{2}-u_{1})}\cdots (n-1)\text{λ}e^{-(n-1)\text{λ}(u_{n-1}-u_{n-2})}\cdot e^{-n\text{λ}(t-u_{n-1})}\\ & =\;{\text{λ}}^{n-1}e^{-n\text{λ}t}(n-1)!\;\exp\left(-\text{λ}\left[\sum_{j=1}^{n-1}j(u_{j}-u_{j-1})-nu_{n-1}\right]\right)\\ & =\;e^{-n\text{λ}t}(n-1)!\;\prod_{j=1}^{n-1}\text{λ}e^{\text{λ}u_{j}}. \end{array}$$

To find ℙ(Z(t)=n|Z(0)=1), we integrate the right side of (2.1) over $0<u_{1}<\cdots <u_{n-1}<t$. Recalling that


$$\int_{0}^{t}\text{λ}e^{\text{λ}u_{1}}du_{1}\;\int_{u_{1}}^{t}\text{λ}e^{\text{λ}u_{2}}du_{2}\;\cdots \int_{u_{n-2}}^{t}\text{λ}e^{\text{λ}u_{n-1}}du_{n-1}=\frac{1}{(n-1)!}{\left(e^{\text{λ}t}-1\right)}^{n-1},$$

and simplifying, we see that


(2.2) $$P(Z(t)=n|Z(0)=1)=e^{-\text{λ}t}(1-e^{-\text{λ}t}{)}^{n-1},\;n=1,2,\ldots .$$

Thus, Z(t) has a geometric distribution with mean and variance given by


$$E(Z(t)|Z(0)=1)=e^{\text{λ}t},\;Var\;(Z(t)|Z(0)=1)=e^{\text{λ}t}(e^{\text{λ}t}-1).$$

If we start with r genera that evolve independently, the total number of species after time t has the negative binomial distribution,


(2.3) P(Z(t)=n∣Z(0)=r)=(n−1r−1)e−rλt(1−e−λt)n−r,n=r,r+1,….

It is worth noting that the time-inhomogeneous case is readily analysed too. Kendall [9] showed if the instantaneous split rate at time u is λ(u), then the number of species $\tilde{Z}(t)$ at time t still has a geometric distribution, with


$$\mathbb{P}(\tilde{Z}(t)=n|\tilde{Z}(0)=1)=e^{-\rho (t)}(1-e^{-\rho (t)}{)}^{n-1},n=1,2,\ldots ,$$

where


$$\rho (t)=\int_{0}^{t}\text{λ}(u)du.$$

Indeed, the process $\tilde{Z}(\cdot )$ is a deterministic time-change of a Yule process with λ=1; that is,


$$\tilde{Z}(t)=Z(\rho (t)),\;t\geq 0.$$

### (b) Yule trees

**Figure 1.**
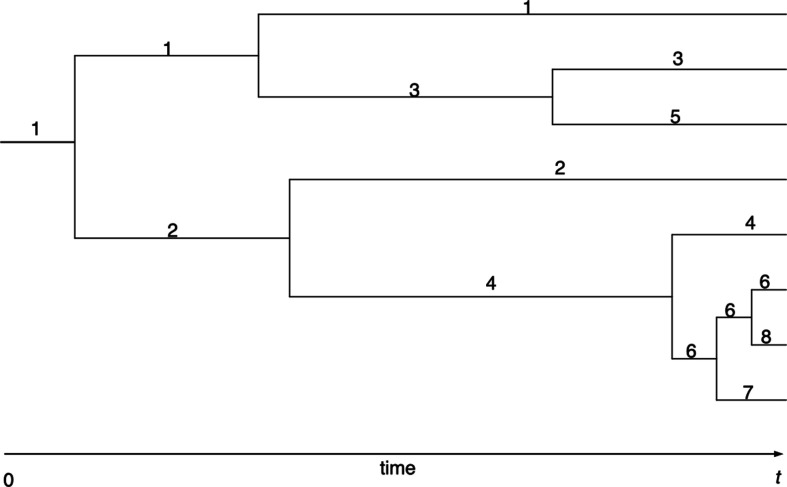
Evolution of a Yule process. Species give rise to new species at constant rate λ. The original species is labelled 1, its first descendant 2, the next new species 3, and so on. Time runs from left to right. This example has eight species alive at time t. The tree topology is that of a bifurcating ultrametric tree.

The evolution of the population is often depicted as a tree with binary splits. Figure 1 illustrates a tree with eight species extant at time t. The figure suggests that new species arise faster and faster, as the split rates would confirm. We can say more though.

From (2.1) and (2.2), we see that the joint density of $U_{1},U_{2},\ldots ,U_{n-1}$ conditional on Z(t)=n is


(2.4) $$\begin{array}{rl} f_{n}(u_{1},\ldots ,u_{n-1}|Z(t)=n)\; & =\;{\text{λ}}^{n-1}(n-1)!\;e^{-n\text{λ}t}e^{\text{λ}\sum u_{j}}/e^{-\text{λ}t}(1-e^{-\text{λ}t}{)}^{n-1}\\ \; & =\;(n-1)!\prod_{j=1}^{n-1}\frac{\text{λ}e^{-\text{λ}(t-u_{j})}}{1-e^{-\text{λ}t}}, \end{array}$$

for $0<u_{1}<\cdots <u_{n-1}<t$. The density in (2.4) may be written in the form


(2.5) $$\begin{array}{lcr} & f_{n}(u_{1},\ldots ,u_{n-1}|Z(t)=n)=(n-1)!\;g(u_{1})\cdots g(u_{n-1}), & \end{array}$$

where g(u) is the probability density


(2.6) $$g(u)=\frac{\text{λ}e^{-\text{λ}(t-u)}}{1-e^{-\text{λ}t}},\;0<u<t.$$

The form of (2.5) identifies the conditional distribution of the (n−1) split times $U_{1},\ldots ,U_{n-1}$ as the distribution of the increasing order statistics of a random sample from the density (2.6).

Properties of the split times follow from standard results concerning order statistics. One immediate application is the simulation of trajectories having precisely n extant species at time t. The naive rejection method would simulate trajectories for time t and keep any that have Z(t)=n. The likely success of this strategy can be judged from (2.2). Simpler is to generate a random sample $V_{1},\ldots ,V_{n-1}$ from the uniform distribution on (0,1), and set


$$S_{i}={\text{λ}}^{-1}\log\left(e^{\text{λ}t}V_{i}+(1-V_{i})\right),\;i=1,2,\ldots ,n-1.$$

$S_{i}$ has density (2.6), and the increasing order statistics of $S_{1},\ldots ,S_{n-1}$ have the required distribution.

## 3. Birth and death processes

### (a) Distributional properties

For ease of exposition, we focus on the time-homogeneous linear processes with rates, from (1.1),


$${\text{λ}}_{n}=n\text{λ},\;\mu_{n}=n\mu ,\;\text{λ}>0,\mu \geq 0.$$

The distribution of the population size Z(t) was found in [3]. Defining


$$p_{1j}\;(t)=P\;(Z(t)=j|Z(0)=1),j=0,1,2,\ldots ,$$

we have for t>0,


(3.1) $$\begin{array}{rl} p_{1m}(t)\; & =\;\left(1-\alpha \left(t\right)\left(1-\beta (t\right)\right)\beta {\left(t\right)}^{m-1},m=1,2,3,...\\ p_{10}(t)\; & =\;\alpha (t), \end{array}$$

where


(3.2) $$\begin{array}{rl} \beta (t)\; & =\;\frac{\text{λ}(e^{(\text{λ}-\mu )t}-1)}{\text{λ}e^{(\text{λ}-\mu )t}-\mu }\;and\;\alpha (t)=\frac{\mu }{\text{λ}}\;\beta (t),\;\text{λ}\neq \mu \\ & =\;\frac{\text{λ}t}{1+\text{λ}t}\;and\;\alpha (t)=\beta (t),\;\text{λ}=\mu . \end{array}$$

Note that


$$p_{10}(t)\to \min\{1,\mu /\text{λ}\}\;as\;t\to \infty ,$$

showing that starting from a single individual, the population goes extinct with probability min{1,μ/λ}. If Z(0)=m, the extinction probability is $\min\{1,(\mu /\text{λ}{)}^{m}\}$, corresponding to the extinction of each population initiated by a single individual.

We record for future use the fact that


(3.3) $$\frac{d\beta (t)}{dt}=\text{λ}(1-\alpha (t))(1-\beta (t)).$$

Many other properties of this model are collected in [10], including the transition functions starting from more than one individual, numerical methods for computing them and a comparison of inference methods based on serial observation of the population size. We will use (3.1) in §4.

### (b) Birth and death trees

**Figure 2.**
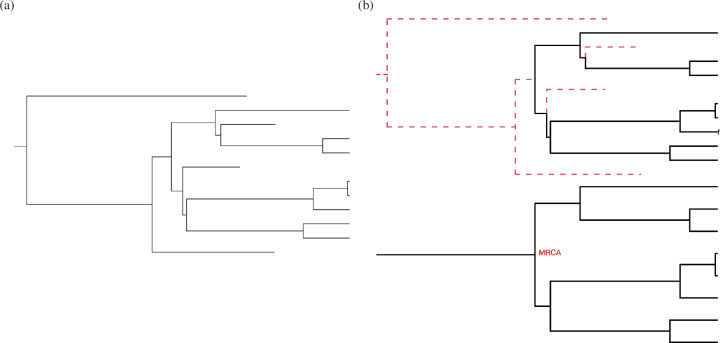
(*a*) A birth-and-death tree starting from a single species at time 0, on the far left. Births result in a bifurcation in the tree and deaths cause the end of a lineage. The event rate when there are n extant lineages is n(λ+μ) and an event in a randomly chosen lineage results in a bifurcation with probability λ/(λ+μ) or the end of that lineage with probability μ/(λ+μ). The population on the far right (b) is composed of extant individuals (here, eight of them). The split times are determined by (3.4). Once the split times are given, the topology of the tree is generated by bifurcating a random existing lineage. (*b*) The top tree highlights the history of species that have died out (red dashed lines). The lower tree is the reconstructed birth–death tree, in which lineages that do not reach the current time at the right of the tree are removed. Extant species are traced back to their MRCA. Trees drawn with help from the R package phytools [11].

Just as for the Yule process tree in figure 1, the ancestral links among the individuals in a birth and death process can be represented as a tree, as shown in figure 2a. Many inferential problems in phylogenetics involve estimating the properties of such a tree based on data such as molecular sequence data collected from the species represented at the tips, and possibly additional information about the ancestral nodes such as sampling dates and fossil occurrences. The BDPs represent just one class of stochastic models used in this endeavour, primarily owing to their tractability.

Some inferential problems involve estimation of the ancestral history of species at the tips, for example, inferring the relatedness among the species. Others involve inference about the branch lengths in the tree. For example, in [12,13] and [14], birth and death processes are used to estimate the time of the most recent common ancestor (MRCA) of extant primates, both with and without additional molecular data from those primates. A general approach to such problems is given in [15]. Bayesian inference for these and similar problems, using Markov chain Monte Carlo methods, are available in BEAST 2.5 [16].

We are often interested in the ancestral history of the extant species, rather than of the species that have gone extinct in the past. The ancestral history of extant species is recorded in their *reconstructed* tree, the study of which was initiated in [17,18]. An example is shown in figure 2. The timing of events in a reconstructed tree depends on the sampling scheme—for example, whether that tree runs for time t, runs until there are n species, or runs until there are n species that have evolved over time t.

To simulate trees under the last scenario starting from a single species at time 0, the split times $U_{1},U_{2},\ldots ,U_{n-1}$ are generated from the increasing order statistics of n−1 independent random variables with probability density


(3.4) $$\begin{array}{rl} g(u)\; & \propto \;\text{λ}P(Z(t-u)=1|Z(0)=1)=\text{λ}(1-\alpha (t-u))(1-\beta (t-u))\\ & =\;\text{λ}(1-\alpha (t-u))(1-\beta (t-u))/\beta (t)\;0<u<t, \end{array}$$

where α(t) and β(t) are given in (3.2) and the normalizing constant follows from (3.3). The distribution function corresponding to (3.4) is


$$G(u)=1-\frac{\beta (t-u)}{\beta (t)},\;0<u<t,$$

so the order statistics for a sample from (3.4) can be found using the probability integral transform. The result in (3.4) can be found in [17,19] and its extension to the inhomogeneous case in [20]. For the Yule process, this result is proved in (2.5) and (2.6). A description of how to simulate the other two scenarios is given in [20], and [21] describes birth-and-death point processes and relates this to coalescent trees.

## 4. Population genetics and ecology

### (a) Population genetics

Among the well-known stochastic models in population genetics is Kingman’s coalescent [22]. It provides a description of the ancestral relationships among a stationary sample of n (haploid) individuals. There are two components to the process: the topology of the binary tree linking the ancestors of the sample backwards in time and the timing of the merges in the tree. The process evolves as follows. Starting from n individuals in the sample, wait a time $T_{n}$ for a randomly chosen pair of individuals to have a common ancestor. At time $T_{n}$, there are n−1 distinct ancestors. Wait a further time $T_{n-1}$ for a randomly chosen pair of the n−1 ancestors to have a common ancestor, resulting in n−2 distinct ancestors at time $T_{n}+T_{n-1}$ in the past. The process continues in this way until at time $T_{n}+\cdots +T_{2}$ the sample is traced back to its MRCA of the sample. The random variables $T_{n},T_{n-1},\ldots ,T_{2}$ are mutually independent and exponentially distributed, with $ET_{j}=2\;/\;j\;(j-1)$, and independent of the topological properties of the ancestral tree of the sample. Figure 2b provides an example coalescent tree, with time read from right to left.

The effects of neutral mutations may be superimposed on the coalescent tree (for example, the infinitely many alleles and infinitely many sites models), the coalescent providing an efficient way to simulate the behaviour of a sample without needing to simulate a larger population from which the sample arose. The effects of variable population size, selection and recombination can also be modelled by variants of the coalescent. Elementary book-length introductions to the theory and applications appear in [23–25].

We will leave the coalescent here and move on to discuss other generative stochastic models that are useful for modelling in ecology (the appearance of new species in a series of specimens), population genetics (the appearance of new alleles from sampled individuals) and molecular evolution (the appearance of Covid variants among sequentially sampled individuals). We will keep the language neutral by referring to the appearance of families initiated by an immigration process, each family evolving according to a common growth model.

Karlin and McGregor [26] described a model with two components: an immigration process describing the arrival of families (for example, a Poisson process or a renewal process) and a process describing the growth of each family (for example, a Yule process, a birth and death process, a branching process or a Markov chain). We consider the special case in which the immigration process is a homogeneous Poisson process of rate θ and families evolve according to a homogeneous BDP as described in §3a.

**Figure 3.**
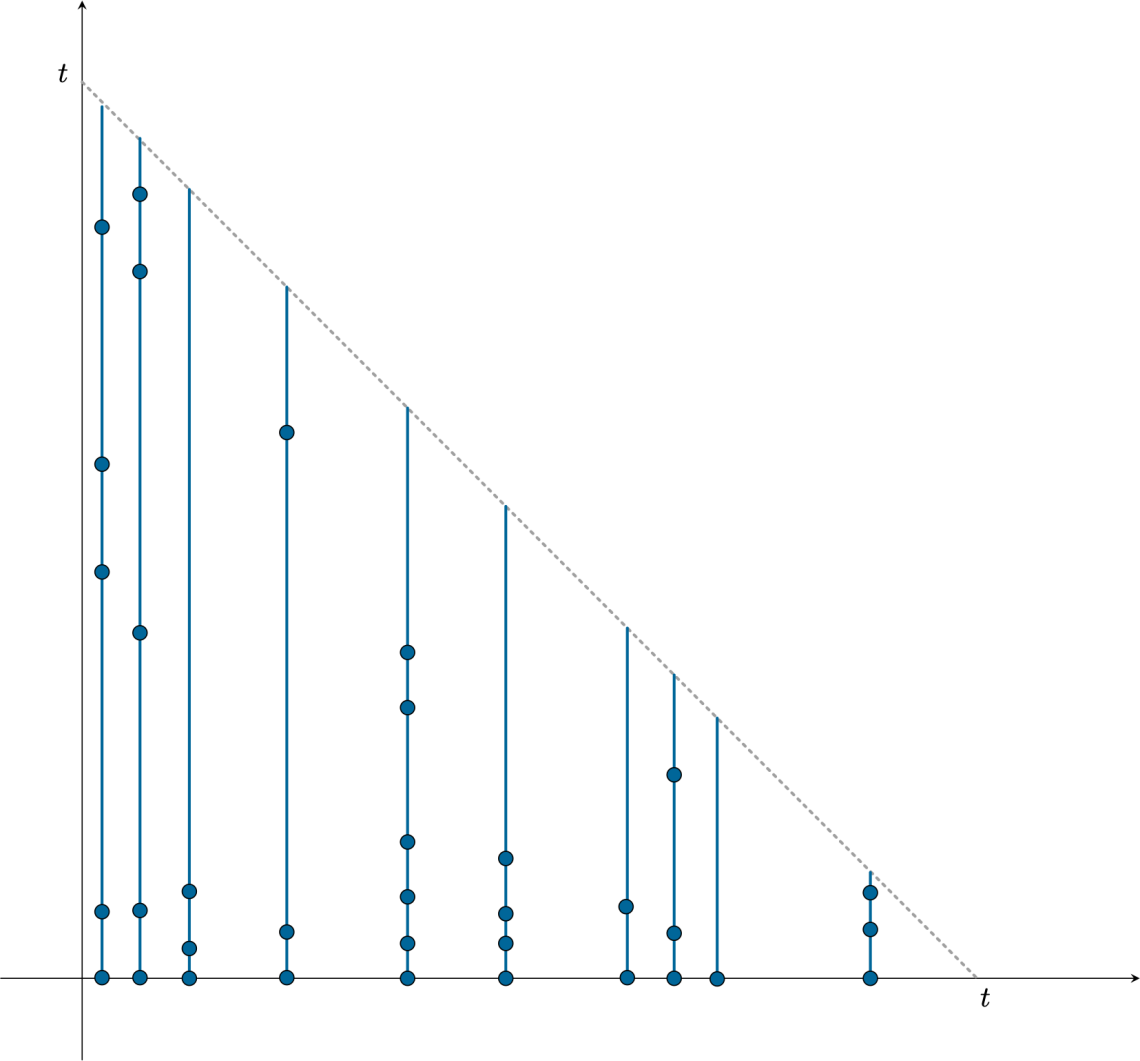
The figure shows how the Poisson marking approach works. Along the *x*-axis the arrival points of immigrants up to time t are shown. The marks on the vertical lines above each such point show the births that have arisen in each family from its arrival up to time t. Each arrival point of the *x*-axis is marked with the number of points on the vertical line up to the diagonal. Counting the number $C_{j}(t)$ with j dots gives $C_{1}=1,C_{2}=1,C_{3}=4,C_{5}=2,C_{6}=1.$ Equation (4.1) gives the distribution of the counts $C_{j}(t)$.


We exploit marked Poisson process arguments to derive a number of basic results. Each family is marked with its size (figure 3). The sets of points with different marks are independent of one another. If $C_{j}(t)$ denotes the number of families marked with a j, then the Poisson Marking Theorem (e.g. [27], §5.2) shows that $C_{j}(t)$ has a Poisson distribution, with

(4.1) $$EC_{j}(t)=\theta \int_{0}^{t}p_{1j}(t-u)du,\;j=0,1,2,\ldots$$

Here, $p_{1j}\;(v)$ is the probability that the growth model has value j at time v, starting from the single immigrant individual at time 0.

The population size at time t is $Z(t)=C_{1}(t)+2C_{2}(t)+\cdots$, while the number of families alive at time t is $F(t)=C_{1}(t)+C_{2}(t)+\cdots$, which is Poisson distributed with mean


$$\sum_{j\geq 1}\theta \int_{0}^{t}p_{1j}(t-u)\;du=\theta \int_{0}^{t}\sum_{j\geq 1}p_{1j}(t-u)\;du=\theta \int_{0}^{t}(1-p_{10}(u))\;du.$$

For the BDP, (4.1) may be calculated explicitly via (3.1) and (3.3) and we obtain


(4.2) $$EC_{j}\;(t)=\frac{\theta }{\text{λ}}\frac{\beta \;(t{)}^{j}}{j},\;j=1,2,\ldots ,$$

where β(t) is given in (3.2), and


(4.3) $$EC_{0}(t)=\log\;\left(e^{\theta t}\;(1-\beta \;(t){)}^{\theta /\text{λ}}\right).$$

The distribution of Z(t) is the well-known negative binomial [9], because its probability-generating function is


$$Es^{Z(t)}=Es^{\sum_{j\geq 1}jC_{j}(t)}=\prod_{j\geq 1}Es^{jC_{j}(t)}=\exp\left(\frac{-\theta }{\text{λ}}\sum_{j\geq 1}\frac{\beta (t{)}^{j}}{j}(1-s^{j})\right)={\left(\frac{1-\beta (t)}{1-\beta (t)s}\right)}^{\theta /\text{λ}},$$

exploiting the independent, Poisson nature of the $C_{j}(t).$ We have


(4.4) P(Z(t)=j∣Z(0)=0)=(j+θ/λ−1j)(1−β(t))θ/λβ(t)j, j=0,1,2,…

and the mean population size at time t is $EZ(t)=\theta (e^{(\text{λ}-\mu )t}-1)/(\text{λ}-\mu )$ if λ≠μ and θt if λ=μ [9].

If we know the number of individuals alive at time t, say Z(t)=n, then the conditional distribution of the counts $C_{1}(t),\ldots ,C_{n}(t)$ has a familiar form [28,29]: for integers $c_{1}\geq 0,c_{2}\geq 0,\ldots ,c_{n}\geq 0$ satisfying $c_{1}+2c_{2}+\cdots +nc_{n}=n$,


(4.5) $$P(C_{1}(t)=c_{1},\ldots ,C_{n}(t)=c_{n}|Z(t)=n)=\frac{n!}{(\theta /\text{λ}{)}_{\left(n\right)}}\;\prod_{j=1}^{n}{\left(\frac{\theta }{\text{λ}j}\right)}^{c_{j}}\frac{1}{c_{j}!},$$

where $x_{\left(n\right)}:=x(x+1)\cdots \left(x+n-1\right)$. The distribution in (4.5) is known in population genetics as the Ewens sampling formula [5], where it gives the distribution of the allele counts in a sample of size n from a neutral, stationary population with mutation parameter θ/λ.

The sizes of families in a BDP may be tracked in order of their appearance, as opposed to being summarized by the number of families of particular sizes. There is an explicit formula for the probability of observing l extant families, the oldest of size $\eta_{1}$, the next oldest of size $\eta_{2}$, …, the youngest of size $\eta_{l}$; see [30] for details. This process is very useful for determining the asymptotic behaviour of the largest families, for example, as t→∞ [30,31].

### (b) Ecology

We will close this section with another look at Fisher’s famous ecological inference problem [6] that arose in studying the sampling of specimens belonging to a collection of species. Fisher argued that the expected number of species $EC_{j}$ found j times in a sample had the form $EC_{j}=\theta \eta^{\;j}/j,\;j=1,2,\ldots$ for η∈(0,1). Letting F denote the number of species found (the number of families in our earlier parlance) and Z the number of specimens (the number of individuals earlier), Fisher noted that


$$\begin{array}{rl} EF\; & =\;E(C_{1}+C_{2}+\cdots )=-\theta \log(1-\eta )\\ EZ\; & =\;E(C_{1}+2C_{2}+\cdots )=\theta \eta /(1-\eta ), \end{array}$$

and he developed a method of moments method for estimating (θ,η) from observations on (F,Z). Fisher’s more challenging problem was to understand the ‘variance of F and Z in parallel samples’.’ Much hinges on the interpretation of the terms, but there are at least two distinct interpretations: we might take (i) two independent samples of size n from replicate populations, or (ii) two consecutive samples from the same population. Let $F_{1}$ and $F_{2}$ denote the number of species found in the first and second samples, respectively. Fisher’s problem then concerns the behaviour of the sample variance of $F_{1}$ and $F_{2}$, the quantity


$$V_{2}=\frac{1}{2}(F_{1}-F_{2}{)}^{2}.$$

In scenario (i), $EV_{2}∼\theta \log n$, whereas in scenario (ii), Fisher claimed that $EV_{2}∼\theta \log2$ in large samples. A recent discussion of the history, together with an exact treatment of a discrete-time multiple sequential sampling case appears in [32].

**Figure 4.**
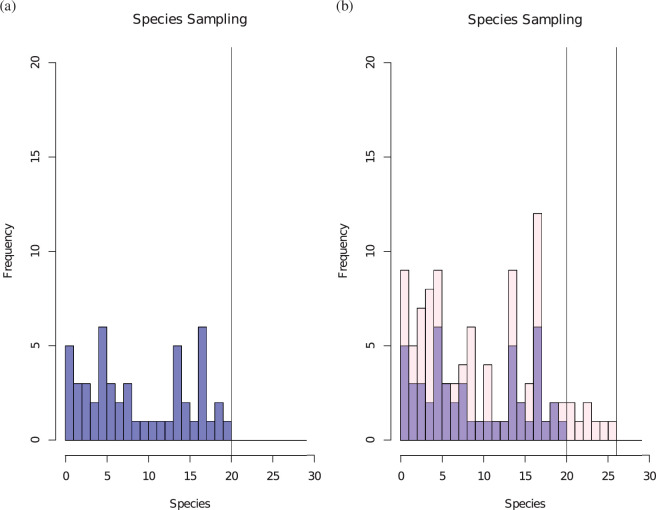
(*a*) The result of classifying 50 specimens into species. The first specimen is of a novel species, the second may be novel or a member of an existing species, and so on as the specimens arrive. There were $F_{1}=20$ species found in the first 50 specimens. (*b*) Categorization of the next 50 specimens. Many will be examples of species found in the first 50 specimens and some will be examples of new species. In this case, eight specimens represented six new species, the other 42 being assigned to 13 existing species; so $F_{2}=13+6=19$. The correlation between $F_{1}$ and $F_{2}$ depends on the nature of the stochastic allocation of specimens to species.

Figure 4 illustrates where one difficulty arises, namely the species detected in different samples. Another difficulty is the apparent lack of an explicit model for the sampling process, and here we follow [33] in using the Yule process with immigration and time scaled so that λ=1, as the generative model.

We define F(a,b) to be the number of families that arrive in (0,a) and have offspring in (a,b),0<a<b . Corollary 1 in [33] shows that


$$F(a,b)\text{ has a Poisson distribution with mean }\theta \;\log(e^{b}-e^{a}+1)),$$

while Corollary 2 in [33] establishes that for 0≤a<b≤c<d,


$$\mathrm{Cov}(F(a,b),F(c,d))=\theta \;\log\left(\frac{\left(e^{b}-e^{a}+1)(e^{d}-e^{c}+1\right)}{e^{d}-e^{c}+e^{b}-e^{a}+1}\right).$$

**Figure 5.**
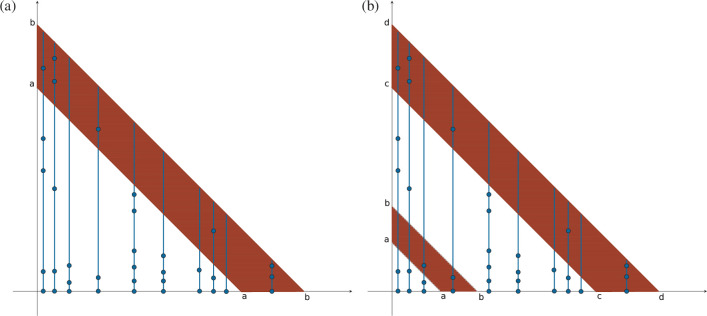
(*a*) Marking of Poisson arrivals in (0,a) that have offspring in (a,b),0<a<b. Here, F(a,b)=4; F has a Poisson distribution. (*b*) Marking of Poisson arrivals in (0,d) that have offspring in (a,b) and (c,d). Here, F((a,b),(c,d))=1 and F also has a Poisson distribution ([33], Theorem 1).

See figure 5 for an illustration of the use of the Poisson Marking Theorem.

These two results allow us to compute the mean of the sample variance of $F(0,t_{1}),F(t_{1},t_{2})$ for any $t_{0}=0<t_{1}<t_{2}$ and we obtain


(4.6) $$EV_{2}=\frac{1}{2}\left\{\theta \log(e^{2t_{2}}e^{-t_{1}}/(e^{t_{2}}-e^{t_{1}}+1))+\theta^{2}\log^{2}(e^{t_{1}}/(e^{t_{2}}-e^{t_{1}}+1))\right\}.$$

To compare this result with the discrete case of consecutive samples of size $n_{1},n_{2}$ considered by Fisher, we need to choose the time points $t_{1},t_{2}$. Recalling that $EZ(t)=\theta (e^{t}-1)$, we can choose the time points in such a way that the expected number of specimens at $t_{1}$ is $n_{1}$, and at $t_{2}$ is $n_{1}+n_{2}$. This results in


$$t_{1}=\log(n_{1}+\theta )-\log\;\theta ,\;t_{2}=\log(n_{1}+n_{2}+\theta )-\log(\theta ),$$

and (4.6) reduces to


(4.7) $$EV_{2}=\frac{1}{2}\left\{\theta \log\left(\frac{(n_{1}+n_{2}+\theta {)}^{2}}{\left(n_{1}+\theta )(n_{2}+\theta \right)}\right)+\theta^{2}\log^{2}\left(\frac{n_{1}+\theta }{n_{2}+\theta }\right)\right\}.$$

When the sample sizes are equal, as envisaged by Fisher, (4.7) reduces to


$$EV_{2}=\theta \log\left(\frac{2n+\theta }{n+\theta }\right),$$

as given in [6, p. 451], and justifying the claim that $EV_{2}∼\theta \log2$ in large samples. [33] provides proofs and generalizations of these results to the case of multiple samples.

## 5. Statistical inference

In this section, we describe a recent advance in ABC that shows great promise for estimating the joint posterior distribution of a set of model parameters. We begin with a brief description of ABC.

ABC was developed to deal with inference problems with intractable likelihoods [34–37]. Imagine a stochastic model with parameter θ (which may be multidimensional) and observed data $D^{obs}$. The aim is to find the posterior


(5.1) $$f(\theta |D^{obs})\propto L(D^{obs}|\theta )\;\pi (\theta ),$$

where L is the likelihood under the model and π the prior for θ.

In most problems, the likelihood in (5.1) is either intractable or very time consuming to compute. ABC offers an alternative that does not make explicit use of the likelihood. Repeat the steps below:

- Generate θ∼π(⋅);
- Generate $D^{sim}$ from the model with parameter θ; and
- Accept θ if $D^{sim}=D^{obs}$

Of course, this naive rejection method fails if the acceptance probability is too small, as will usually be the case in practice. One approach to remedy this problem is to summarize the data D via a set of summary statistics, S(D), accepting those θ-values for which the distance $\rho (S(D^{sim}),S(D^{obs}))$ is less than some prescribed cutoff, ϵ.

The art in this approach, and a potential stumbling block, is the choice of summary statistics S, the metric ρ and the cutoff ϵ. For example, one would like to identify summary statistics that contain as much information about θ as possible. From its introduction in the late 1990s, ABC has developed into a very useful tool in the statistician’s arsenal and there are many approaches motivated by the above discussion, including Markov chain Monte Carlo and sequential Monte Carlo. The *Handbook of approximate Bayesian computation* [38] illustrates the methods available up until 2018.

A substantial contribution to the practice of ABC was made by Raynal *et al*. [39]. The authors suggested the use of random forest regression as a way to circumvent issues with choice of summary statistics, the metric and the tolerance. The ABC random forests (ABC-RF) method provides a way to infer the marginal distribution of each parameter, one at a time, and is particularly robust to the choice of summary statistics. A convenient R package is available as ABC–RF [39]. The method uses a reference table composed of summary statistics (the covariates) from repeated simulations of the model generated from parameter values selected from the prior (the response variable). The posterior is formed by random forest regression using the reference table.

### (a) A birth–death process example

BDPs are often used to model aspects of cell division in tumours, for example. For a recent study assessing the roles of copy number aberrations and selection, see [40]. Here, we illustrate an extension of ABC–RF to the multivariate parameter setting [41] in a simplified stochastic model of somatic evolution.

Cells age independently for an exponential amount of time with parameter λ. At the next event, a cell either dies or divides, the division probability being p∈(0,1). The process is grown from a single cell until there are a total of N cells, and a random sample of size n is chosen from the population for analysis. Simulations of the process that die out before reaching N cells are ignored. The data come from the effects of mutation occurring on the branches of the ancestral tree of the sample, including truncal mutations that arise in every sampled cell; we assume these occur according to an infinitely many-sites model of rate θ. The data are given by the site frequency spectrum (SFS) $f_{1},f_{2},\ldots ,f_{n}$, in which $f_{j}$ is the number of mutations appearing in j of the cells. The total number of mutations is $S=f_{1}+f_{2}+\cdots +f_{n}$. The aim is to approximate the posterior distribution of the parameters (λ,p,θ).

In this example, we fix the rate λ=1, and we bin the SFS by considering the proportions $b_{1},b_{2},\ldots ,b_{10}$ of the S mutations that occur in fractions (0,0.1],(0.1,0.2],…,(0.9,1.0] of the sample of size n. The values of S, $\left(b_{1},\ldots ,b_{9}\right)$ and the number of truncal mutations form the summary statistics of interest.

The example illustrates the use of Ćevid *et al*.’s distributional random forest regression method [42] adapted for use as ABC distributional random forests (ABC–DRF) as in [41]. A reference table is constructed as earlier, but now the response variable is multidimensional, in our case (p,θ), and the resulting method estimates the *joint* posterior distribution of the parameters. This approach considerably enhances the utility of RF-based methods, as it deals effectively with multidimensional parameters.

**Figure 6.**
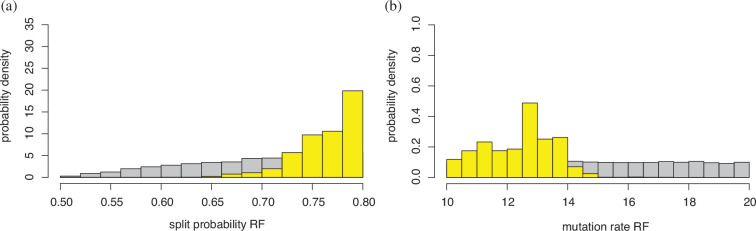
Results for marginal posteriors from ABC random forests (ABC–RF). The prior is in grey, the posterior in yellow. (a) The prior is not uniform, as simulations that do not reach 1000 cells are discarded, thereby biassing the split probability p upwards. (b) The prior is indeed uniform, as there is no implicit conditioning. The posteriors cover the actual values of θ=11.07 and p=0.722.

**Figure 7.**
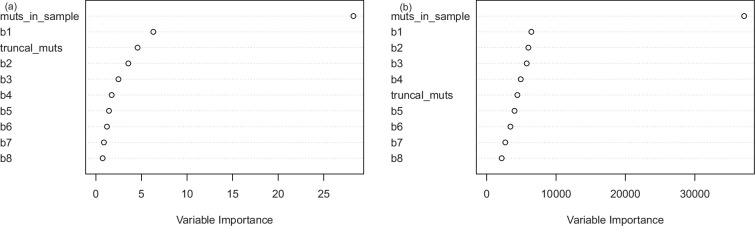
Variable importance plot from ABC–RF. (a) for split probability p, (b) for the mutation rate θ. As expected, the observed number of mutations is the most important covariate, followed by $b_{1}$. The number of truncal mutations is also an important variable for determining p.

**Figure 8.**
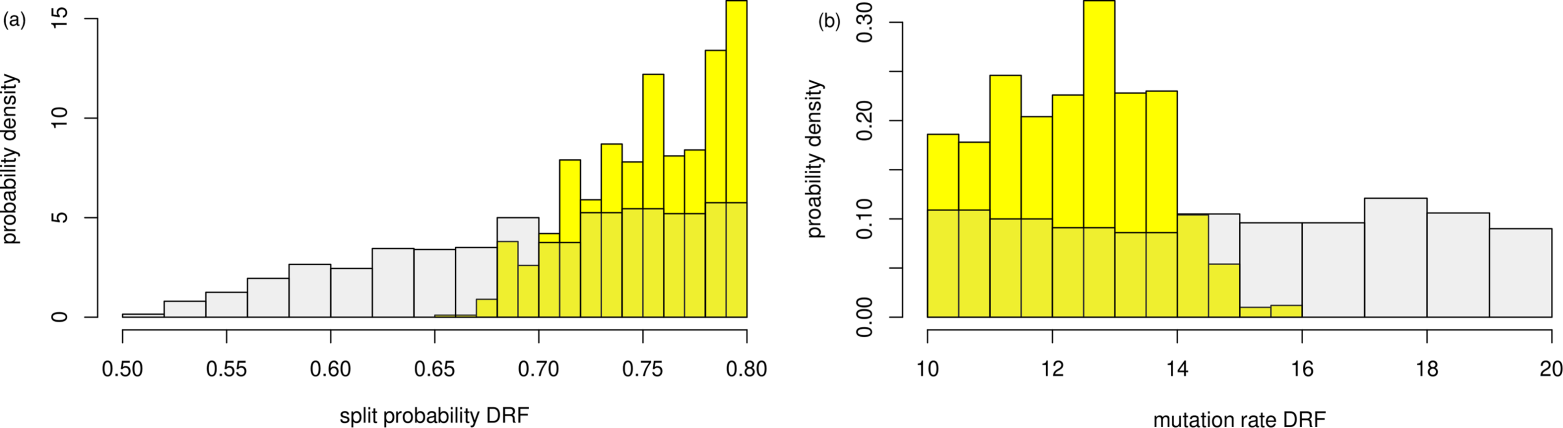
Results for marginal posteriors from ABC–DRF. The prior is in grey, the posterior in yellow. (a) the split rate p; (b) the mutation rate θ. The posteriors cover the actual values of θ=11.07 and p=0.722.

**Figure 9.**
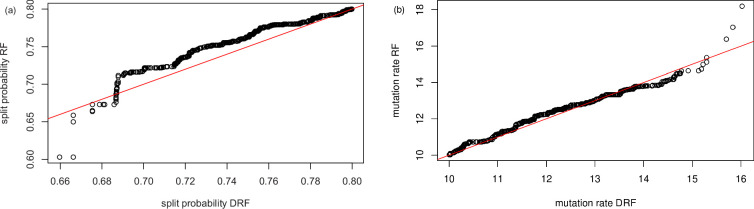
Q–Q plot to compare the marginal posteriors of (a) the split probability p and (b) the mutation rate θ. The distributions are broadly comparable.

**Figure 10.**
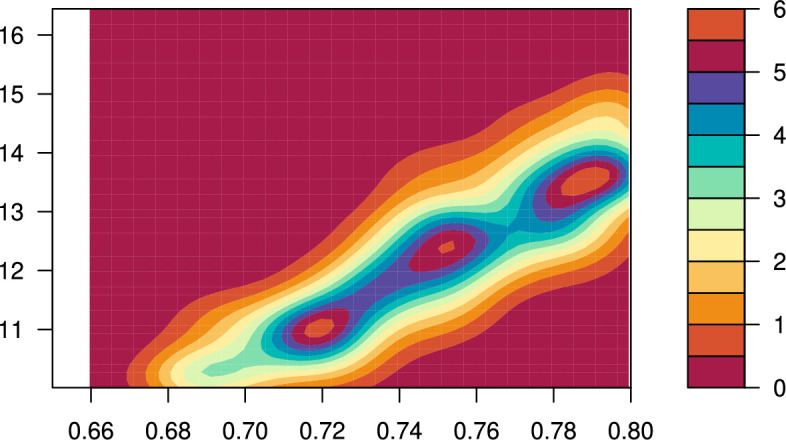
Joint density contours from ABC–DRF. x-axis, split probability p; y-axis, mutation rate θ.

In the following illustration, the prior for θ is uniform on (10,20), and for p it is uniform on (0.5,0.8); the reference table is formed of 10 000 sets of values of $S,\left(b_{1},b_{2},\ldots ,b_{9}\right)$ and the number of truncal mutations observed. Below we compare the results for ABC–RF and ABC–DRF inference, using as the observed data a single run from an independent test table of size 1. The observed values of the summary statistics are $S=9573,(b_{1},\ldots ,b_{9})=(0.985,0.007,0.002,0.001,0.002,0,0,0,0)$ and no truncal mutations were observed. The data were generated with θ=11.07,p=0.722. The results are summarized in figures 6–10.

This example was chosen to illustrate how ABC–DRF adds a useful component to ABC methods based on random forests. The same approach may be used to study the posterior distribution of other features of the model, such as the time to the MRCA of the sample or the age of the cell population. Recent developments of sequential Monte Carlo ABC–DRF [43] may prove useful for this.

## Data Availability

This article has no additional data.
